# Complete Genome Sequence of a Salmonella enterica subsp. *enterica* Serovar Tennessee Strain from Tahini

**DOI:** 10.1128/mra.00407-22

**Published:** 2022-07-27

**Authors:** Olga Schlund, Andre Göhler, Maria Borowiak, Carlus Deneke, Matthias Fischer, Marina C. Lamparter

**Affiliations:** a German Federal Institute for Risk Assessment, Department of Biological Safety, Food Microbiology, Host-Pathogen Interactions, Berlin, Germany; b Humboldt University of Berlin, Life Science Faculty, Institute for Biology, Berlin, Germany; c German Federal Institute for Risk Assessment, Department of Biological Safety, National Study Centre for Sequencing in Risk Assessment, Berlin, Germany; University of Maryland School of Medicine

## Abstract

Salmonella sp. infections are associated with contaminated low-moisture foods (with high fat content) with increasing frequency. Here, we report the complete genome sequence of Salmonella enterica subsp. *enterica* serovar Tennessee, which was isolated from tahini (a paste made from ground sesame seeds) purchased at a local retailer in Berlin, Germany.

## ANNOUNCEMENT

Recent transnational outbreaks of various Salmonella serovars associated with tahini sesame paste highlight the issue of Salmonella contamination of processed sesame food (1, 2). In a study analyzing nine different tahini products purchased in Berlin, Germany, we found one that was positive for Salmonella, and we isolated (3) Salmonella enterica subsp. *enterica* Tennessee. Briefly, 25 g of tahini was mixed with 225 mL buffered peptone water and incubated for 19 h at 37°C. On modified semisolid Rappaport-Vassiliadis (MSRV) agar, 100 μl was incubated for 24 h at 41.5°C. Cell material from MSRV agar was plated on xylose-lysine-desoxycholate (XLD) agar and incubated for 22 h at 37°C. Black colonies were confirmed as Salmonella spp. using matrix-assisted laser desorption ionization–time of flight mass spectrometry (MALDI-TOF MS) (Bruker Daltonics). Serological typing was performed according to the White-Kaufmann-Le Minor scheme using standard reagents (Sifin Diagnostics GmbH, Berlin, Germany) (4).

For isolation of genomic DNA (gDNA) used for both short-read Illumina sequencing and long-read Oxford Nanopore Technologies (ONT) sequencing, one single colony was enriched in lysogeny broth for 18 h at 37 ± 1°C. The PureLink gDNA minikit (Invitrogen, Carlsbad, CA, USA) was used for gDNA isolation.

The library for short-read sequencing was prepared using the DNA preparation (M) tagmentation kit (Illumina, San Diego, CA, USA). The library was sequenced on the Illumina NextSeq benchtop sequencer using the NextSeq 500/550 midoutput kit v2.5 (300 cycles; Illumina) in 2 × 149-bp cycles. The short-read sequence data were trimmed using fastp v0.19.5 (5). Trimming resulted in 1,666,364 high-quality reads (82.8% [quality scores of ≥Q30]).

The library for the MinION platform (ONT, Oxford, UK) was prepared using the rapid barcoding kit SQK-RBK004 (ONT). The DNA isolation and sequencing kits were used according to the instructions of the manufacturer.

The MinION library was sequenced for 24 h on an ONT MinION Mk1C device (MinKNOW v20.03.5, including Guppy base caller v3.4.8) using a Flongle adapter and a FLO-FLG001 Flongle flow cell. The reads obtained were trimmed using Porechop v0.2.3 (https://github.com/rrwick/Porechop), filtered using NanoFilt v2.7.1, and quality checked using NanoStat v1.4.0 (6). Trimming and filtering resulted in 30,938 reads, with a read *N*_50_ value of 10,167 bp and 71.6% of bases reaching quality scores above 10.

To assemble and circularize the genome sequence, short- and long-read data sets were subjected to the hybrid assembler Unicycler v0.4.8 including Pilon v1.23 (7–9).

The assembly resulted in a circular bacterial chromosome (start gene, *dna*A) and a circular ColRNAI_1 plasmid sequence (Table 1). The overall G+C content of the genome sequence was 52.2%. For annotation, NCBI PGAP v6.0 was used (10).

**TABLE 1:** Summary of determined genetic features of *S*. Tennessee strain 21-SA00318-0

Feature^*a*^	Result(s)	Tool(s), database(s), and options^*b*^
Serovar	Salmonella enterica subsp. *enterica* serovar Tennessee	Sistr_cmd v1.1.1 (20)
MLST	ST319	MLST v2.19.0, PubMLST database (18, 19)
Chromosome size (bp)	4,825,110	
Plasmid type	ColRNAI_1 plasmid	ABRicate v1.0.1, Center for Genomic Epidemiology database, PlasmidFinder, Platon v1.4.0 (mode: accuracy, –verbose) (14–16)
Plasmid size (bp)	3,967	
Virulence determinants		SPIFinder v2.0, ABRicate v1.0.1, VFDB (minicov/minid: 80) (16, 17, 21)
No. of SPIs	7 (SPI-1 to SPI-5, SPI-8, and SPI-9)	
No. of virulence genes	102	
Virulence genes located on SPIs^*c*^	SPI-1: *invH*, *invFGEABCIJ*, *spaOPQRS*, *sicA*, *sipBCDA*, *sicP*, *sptP*, *prgHIJK*, *orgABC*, and *avrA*; SPI-2: *ssaUTSRQPONVMLK*, *ssaJIHG*, *sseGF*, *sscB*, *sseEDC*, *sscA*, *sseBA*, and *ssaEDCB*; SPI-3: *mgtCB* and *misL*; SPI-5: *sopB* and *pipB*	
Other virulence genes	Genes associated with, e.g., adhesion (e.g., *fim* and *lpf*), curli (*csg*), effectors (e.g., *sif*, *slrP*, *sop*, *sse*, and *ste*), iron transport (*ent* and *fep*), and resistance (*mig-14*)	
Antibiotic resistance factors	*mdsAB*	NCBI AMRFinderPlus v3.10.1 (–nucleotide –O Salmonella –ident_min −1 coverage_min 0.5) (12, 13)
Stress resistance factors	*golST*	NCBI AMRFinderPlus v3.10.1 (–nucleotide –O Salmonella –ident_min −1 coverage_min 0.5) (12, 13)

a The virulence and resistance genes listed here were located on the chromosome. MLST, multilocus sequence typing.

b Default parameters were used for all tools unless otherwise specified.

c Contribution was confirmed by visualization of the chromosome annotation in Geneious Prime v2020.2.2 (22).

For further analysis of the genome sequence, the BakCharak pipeline vv2.1.0 was used (11). The pipeline includes modules (tools, databases, and options) (Table 1) for identifying antimicrobial resistance genes, plasmids, and virulence factors and for predicting sequence types (STs) and serotypes (12–20). For screening of Salmonella pathogenicity islands (SPIs), SPIFinder2.0 was used (21, 22). Default parameters were used for all tools unless otherwise specified.

### Data availability.

Sequencing raw reads were deposited in the NCBI Sequence Read Archive (SRA) (accession numbers SRR17858527 [ONT data] and SRR17858528 [Illumina data]). The complete genome sequence of 21-SA00318-0 is available at NCBI (GenBank accession numbers CP091878 [chromosome], CP091879 [plasmid], and GCF_022162985.1 [latest]). All data are encompassed under BioProject accession number PRJNA802760.

## References

[1] European Food Safety Authority. 2021. Multi-country outbreak of multiple *Salmonella enterica* serotypes linked to imported sesame-based products. EFSA Support Publ 18:6922E. doi:10.2903/sp.efsa.2021.EN-6922.

[2] Meinen A, Simon S, Banerji S, Szabo I, Malorny B, Borowiak M, Hadziabdic S, Becker N, Luber P, Lohr D, Harms C, Plenge-Bönig A, Mellou K, Mandilara G, Mossong J, Ragimbeau C, Weicherding P, Hau P, Dědičová D, Šafaříková L, Nair S, Dallman TJ, Larkin L, McCormick J, De Pinna E, Severi E, Kotila S, Niskanen T, Rizzi V, Deserio D, Flieger A, Stark K. 2019. Salmonellosis outbreak with novel *Salmonella enterica* subspecies *enterica* serotype (11:z41:e,n,z15) attributable to sesame products in five European countries, 2016 to 2017. Euro Surveill 24:6–14. doi:10.2807/1560-7917.ES.2019.24.36.1800543. PMC6737830 31507266

[3] International Organization for Standardization. 2020. Microbiology of the food chain - Horizontal method for the detection, enumeration and serotyping of *Salmonella* - Part 1: Detection of *Salmonella* spp. (ISO 6579-1:2017 + Amd.1:2020); International Organization for Standardization, Geneva, Switzerland. https://www.iso.org/standard/56712.html and https://www.iso.org/standard/76671.html.

[4] Grimont PA, Weill F-X. 2007. Antigenic formulae of the *Salmonella* serovars, 9th ed. WHO Collaborating Centre for Reference and Research on *Salmonella*, Paris, France.

[5] Chen S, Zhou Y, Chen Y, Gu J. 2018. fastp: an ultra-fast all-in-one FASTQ preprocessor. Bioinformatics 34:i884–i890. doi:10.1093/bioinformatics/bty560. 30423086 PMC6129281

[6] De Coster W, D'Hert S, Schultz DT, Cruts M, Van Broeckhoven C. 2018. NanoPack: visualizing and processing long-read sequencing data. Bioinformatics 34:2666–2669. doi:10.1093/bioinformatics/bty149. 29547981 PMC6061794

[7] Walker BJ, Abeel T, Shea T, Priest M, Abouelliel A, Sakthikumar S, Cuomo CA, Zeng Q, Wortman J, Young SK, Earl AM. 2014. Pilon: an integrated tool for comprehensive microbial variant detection and genome assembly improvement. PLoS One 9:e112963. doi:10.1371/journal.pone.0112963. 25409509 PMC4237348

[8] Wick RR, Judd LM, Gorrie CL, Holt KE. 2017. Unicycler: resolving bacterial genome assemblies from short and long sequencing reads. PLoS Comput Biol 13:e1005595. doi:10.1371/journal.pcbi.1005595. 28594827 PMC5481147

[9] Wick RR, Judd LM, Gorrie CL, Holt KE. 2017. Completing bacterial genome assemblies with multiplex MinION sequencing. Microb Genom 3:e000132. doi:10.1099/mgen.0.000132. 29177090 PMC5695209

[10] Tatusova T, DiCuccio M, Badretdin A, Chetvernin V, Nawrocki EP, Zaslavsky L, Lomsadze A, Pruitt KD, Borodovsky M, Ostell J. 2016. NCBI prokaryotic genome annotation pipeline. Nucleic Acids Res 44:6614–6624. doi:10.1093/nar/gkw569. 27342282 PMC5001611

[11] Deneke C. 2021. BakCharak: bacterial characterization pipeline. https://gitlab.com/bfr_bioinformatics/bakcharak.

[12] Feldgarden M, Brover V, Haft DH, Prasad AB, Slotta DJ, Tolstoy I, Tyson GH, Zhao S, Hsu C-H, McDermott PF, Tadesse DA, Morales C, Simmons M, Tillman G, Wasilenko J, Folster JP, Klimke W. 2019. Validating the AMRFinder tool and resistance gene database by using antimicrobial resistance genotype-phenotype correlations in a collection of isolates. Antimicrob Agents Chemother 63:e00483-19. doi:10.1128/AAC.00483-19. 31427293 PMC6811410

[13] Feldgarden M, Brover V, Gonzalez-Escalona N, Frye JG, Haendiges J, Haft DH, Hoffmann M, Pettengill JB, Prasad AB, Tillman GE, Tyson GH, Klimke W. 2021. AMRFinderPlus and the reference gene catalog facilitate examination of the genomic links among antimicrobial resistance, stress response, and virulence. Sci Rep 11:12728. doi:10.1038/s41598-021-91456-0. 34135355 PMC8208984

[14] Carattoli A, Zankari E, García-Fernández A, Voldby Larsen M, Lund O, Villa L, Møller Aarestrup F, Hasman H. 2014. In silico detection and typing of plasmids using PlasmidFinder and plasmid multilocus sequence typing. Antimicrob Agents Chemother 58:3895–3903. doi:10.1128/AAC.02412-14. 24777092 PMC4068535

[15] Schwengers O, Barth P, Falgenhauer L, Hain T, Chakraborty T, Goesmann A. 2020. Platon: identification and characterization of bacterial plasmid contigs in short-read draft assemblies exploiting protein sequence-based replicon distribution scores. Microb Genom 6:mgen000398. doi:10.1099/mgen.0.000398. PMC7660248 32579097

[16] Seemann T. 2020. Abricate. https://github.com/tseemann/abricate.

[17] Chen L, Zheng D, Liu B, Yang J, Jin Q. 2016. VFDB 2016: hierarchical and refined dataset for big data analysis–10 years on. Nucleic Acids Res 44:D694–D697. doi:10.1093/nar/gkv1239. 26578559 PMC4702877

[18] Seemann T. 2020. MLST. https://github.com/tseemann/mlst.

[19] Jolley KA, Maiden MCJ. 2010. BIGSdb: scalable analysis of bacterial genome variation at the population level. BMC Bioinformatics 11:595. doi:10.1186/1471-2105-11-595. 21143983 PMC3004885

[20] Yoshida CE, Kruczkiewicz P, Laing CR, Lingohr EJ, Gannon VP, Nash JH, Taboada EN. 2016. The *Salmonella In Silico* Typing Resource (SISTR): an open web-accessible tool for rapidly typing and subtyping draft *Salmonella* genome assemblies. PLoS One 11:e0147101. doi:10.1371/journal.pone.0147101. 26800248 PMC4723315

[21] Roer L, Hendriksen RS, Leekitcharoenphon P, Lukjancenko O, Kaas RS, Hasman H, Aarestrup FM. 2016. Is the evolution of *Salmonella enterica* subsp. *enterica* linked to restriction-modification systems? mSystems 1:e00009-16. doi:10.1128/mSystems.00009-16. PMC5069764 27822532

[22] Schmidt H, Hensel M. 2004. Pathogenicity islands in bacterial pathogenesis. Clin Microbiol Rev 17:14–56. doi:10.1128/CMR.17.1.14-56.2004. 14726454 PMC321463

